# 1103. Endemic Coronavirus Infections in a US Cohort of Children from Birth to 4 Years

**DOI:** 10.1093/ofid/ofad500.076

**Published:** 2023-11-27

**Authors:** Daniel C Payne, Ardythe L Morrow, Shannon C Conrey, Meredith L McMorrow, Monica McNeal, Liang Niu, Allison Burrell, Elizabeth P Schlaudecker, Claire Mattison, Rachel M Burke, Emily A DeFranco, Zheyi Teoh, Jens Wrammert, Lydia J Atherton, Natalie J Thornburg, Mary A Staat

**Affiliations:** US Centers for Disease Control and Prevention, Atlanta, GA; University of Cincinnati College of Medicine, Cincinnati, Ohio; University of Cincinnati College of Medicine, Cincinnati, Ohio; CDC/NCIRD/CORVD/SPB, Atlanta, GA; Cincinnati Children's Hospital Medical Center, Cincinnati, Ohio; University of Cincinnati, Cincinnati, Ohio; Cincinnati Children's Hospital and Medical Center, Cincinnati, Ohio; Cincinnati Children's Hospital Medical Center, Cincinnati, Ohio; Centers for Disease Control, Atlanta, Georgia; Bill & Melinda Gates Foundation, Seattle, WA; Cincinnati Children’s Hospital Medical Center, Division of Infectious Diseases, University of Cincinnati College of Medicine, Cincinnati, Ohio; Cincinnati Children's Hospital Medical Center, Cincinnati, Ohio; Emory University, Decatur, Georgia; Centers for Disease Control, Atlanta, Georgia; Centers for Disease Control and Prevention, Atlanta, Georgia; Cincinnati Children’s Hospital Medical Center, Cincinnati, Ohio

## Abstract

**Background:**

Endemic coronaviruses (“CoVs”) OC43, HKU1, NL63 and 229E are “common cold viruses” related to SARS-CoV-2, but their natural histories are poorly understood. We documented endemic CoV infections in a prospective cohort of US infants and children to determine the extent to which natural infection protects against subsequent homotypic and heterotypic endemic CoV infections and SARS-CoV-2 infections.

**Methods:**

Cincinnati mother-child pairs were enrolled in the third trimester of pregnancy in 2017-18 and children were followed from birth to 4 years with weekly collection of mid-turbinate nasal swabs. Blood was collected at 6 weeks; 6, 12, 18, 24 months; and annually thereafter. Mothers reported on socio demographics, risk factors, and the child’s weekly symptoms. Medical visits were documented from pediatric care providers.

CoV infections were followed for the first 4 years of life (focusing on the most compliant subset of 116 children having >70% weekly sample collection). Infections were identified through nasal swabs tested using a RT-PCR multiplex pathogen panel, and by serum IgG responses using a validated kit at CDC and interpreted using classification and regression tree (CART) analysis.

**Results:**

We detected 398 endemic CoV infections over 317.5 child-years of follow-up (1.1 infections/child-year). Endemic beta-coronaviruses, OC43 and HKU1, were associated with statistically significant homotypic protection (77% and 84%, respectively) after a single infection. Similarly protective homotypic associations (73%) were elicited by NL63, the dominant alpha-coronavirus, after two infections. 229E infections were uncommon. No heterotypic protective association was found for any of the endemic CoVs or for SARS-CoV-2 infections from June 2020 to Nov 2021. The majority of endemic CoVs and SARS-CoV-2 infections were asymptomatic, but this proportion varied by CoV strain. Symptomatic infections were mild for all CoV strains with no hospitalizations.

**Conclusion:**

Natural infection resulted in homotypic immunity but not heterotypic immunity against other CoVs to the 4^th^ birthday. Children were not protected against SARS-CoV-2 by prior endemic CoV infections. CoV infections in these young children were largely asymptomatic or mild.

**Disclosures:**

**Elizabeth P. Schlaudecker, MD, MPH**, Pfizer: Grant/Research Support|Sanofi Pasteur: Advisor/Consultant **Mary A. Staat, MD, MPH**, CDC: Grant/Research Support|Cepheid: Grant/Research Support|Merck: Grant/Research Support|NIH: Grant/Research Support|Pfizer: Grant/Research Support|Up-To-Date: Honoraria

